# Spaceflight redefines ageing‐associated microbiota

**DOI:** 10.1002/imt2.70023

**Published:** 2025-03-28

**Authors:** Yuan Sun, Sai Liu, Long Chen, Zheng Zhou, Mengyu Ma, Jinran Li, Yi Lu, Yiting Shi, Tingting Yao, Ruizhi Feng, Qiulun Lu, Fatimah Qassadi, Philip M. Williams, Tanya M. Monaghan, Guangji Wang, Zheying Zhu, Xinuo Li

**Affiliations:** ^1^ State Key Laboratory of Natural Medicines China Pharmaceutical University Nanjing China; ^2^ Department of Computer Science RWTH Aachen University Aachen Germany; ^3^ State Key Laboratory of Reproduction Medicine and Offspring Health Nanjing Medical University Nanjing China; ^4^ School of Pharmacy The University of Nottingham Nottingham UK; ^5^ NIHR Nottingham Biomedical Research Centre University of Nottingham Nottingham UK; ^6^ Nottingham Digestive Disease Centre, School of Medicine University of Nottingham Nottingham UK

## Abstract

Spaceflight reshapes microbiota and immune function, mitigating some ageing effects while accelerating immune aging, revealing crucial insights for astronaut health and longevity in space missions.

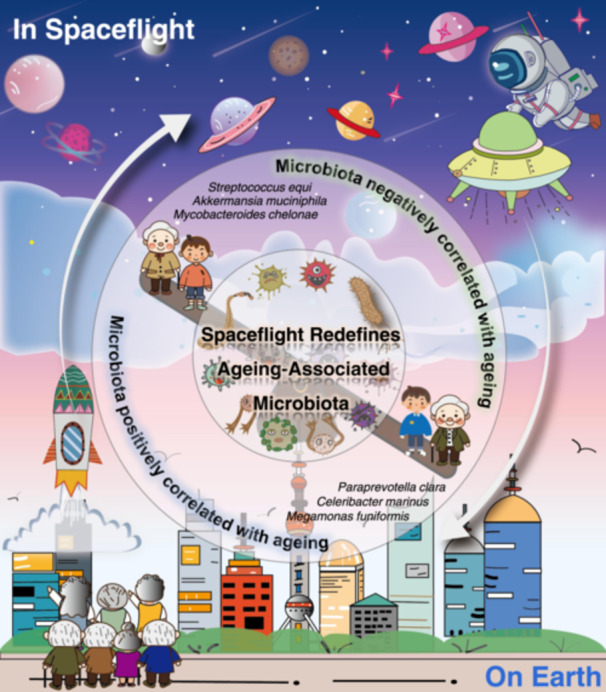

To the Editor,

Space exploration stands as one of humanity's greatest scientific and technological achievements. As advancements lead toward a future where space travel may become more common, understanding the physiological and biological impacts of space on human bodies becomes increasingly important. The extreme conditions of space, including microgravity, increased radiation, and prolonged isolation, present immediate challenges to astronaut health with long‐term implications for human well‐being [1, 2].

The impact of spaceflight on the human immune system, showing inflammatory responses and immune dysregulation, is well‐documented [3, 4, 5]. Additionally, microbiota from various body sites, including the gut, oral cavity, and skin, play important roles in modulating immune responses and influencing the ageing process [6, 7, 8]. Spaceflight‐induced changes in microbiota composition may alter these processes and potentially influence ageing in astronauts [9], though the magnitude and mechanisms of these effects have yet to be fully elucidated. This study addresses this gap by investigating how spaceflight alters microbiota composition and gene expression in relation to ageing.

We integrated metagenomic and metatranscriptomic analyses to explore microbiota changes across body sites during ageing and assess how spaceflight modifies these processes. Our goal was to explore the spaceflight‐induced modulation of microbiota abundance and gene expression across multiple sites in the body and identify potential microbial targets for intervention. These findings aim to inform strategies for promoting healthy ageing and mitigating the health impacts of space travel.

## RESULTS

### Immune system alterations in ageing and spaceflight

**Figure 1:**
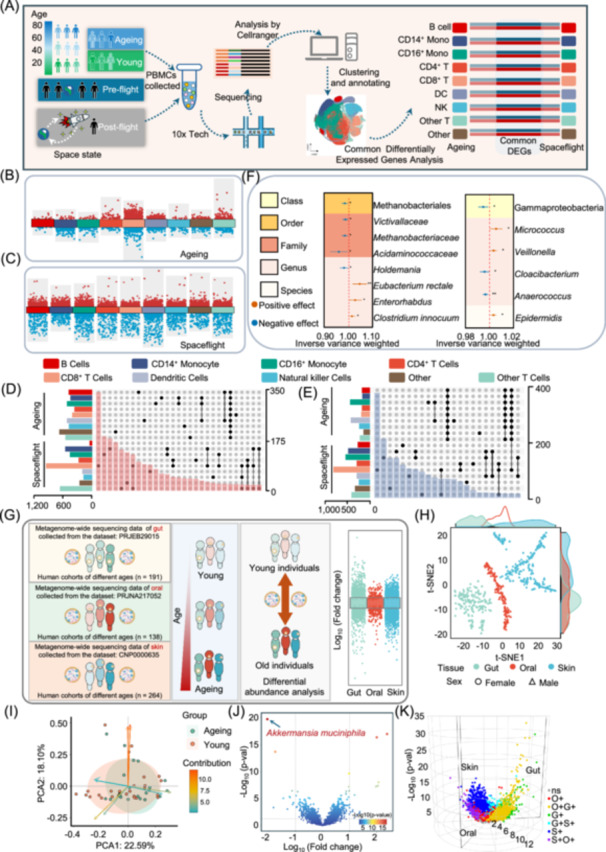
Spaceflight alters the immune function of astronauts in a manner similar to natural ageing. (A) Schematic representation depicting the experimental design and single‐cell analysis of human peripheral blood mononuclear cell (PBMC) samples. (B) Volcano plots depicting differentially expressed genes (DEGs) in major immune cell subsets related to the ageing. (C) Volcano plots depicting DEGs in major immune cell subsets related to the post‐spaceflight. (D) UpSet plots displaying the overlap and distribution of upregulated DEGs between ageing and post‐spaceflight. (E) UpSet plots displaying the overlap and distribution of downregulated DEGs between ageing and post‐spaceflight. (F) Forest plot illustrating causal effects of gut (left) and skin (right) microbiota on longevity. (G) Data collection and analytical approach of metagenomics. (H) t‐SNE plots illustrating the distribution patterns of samples for each group. (I) Principal component analysis (PCA) of gut microbiota in younger and older individuals. (J) Volcano plot depicting gut microbial alterations between younger and older individuals. The marked microbiota are the ones that show the most significant changes in the older group. *Akkermansia muciniphila* has been widely reported to regulate the immune system. (K) 3D volcano plot displaying microbial alterations across three different tissues between younger and older individuals.

To investigate immune function alterations induced by spaceflight and those occurring during ageing, we first analyzed peripheral blood single‐cell RNA sequencing (scRNA‐seq) data from a cohort of ageing individuals (Figure 1A, Table S1). Clustering and annotation revealed nine distinct cell populations (Figure S1A–C), with CD4⁺ T cells being the most abundant (Figure S2A–C). We further analyzed scRNA‐seq data from astronauts' peripheral blood post‐spaceflight (Figure S2D). Differential gene expression analysis showed upregulation of several IL‐6‐related genes both in ageing and post‐spaceflight samples (Figure 1B,C, Tables S2–S3). Additionally, proteomic data revealed increased levels of age‐related inflammatory markers, such as IL‐6 and MCP‐1, in the astronauts' serum (Figure S1D).

To reveal the biological changes in the astronauts after spaceflight, we performed Gene Ontology (GO) enrichment analysis of differentially expressed genes (DEGs) from the ageing cohort revealed significant enrichment in immune‐related pathways (Figure S2E,F). The integrated analysis identified shared DEGs between the ageing process and pre‐ and post‐spaceflight samples (Figure 1D,E), with these genes predominantly enriched in immune‐related pathways (Figure S1E). These findings suggest that spaceflight alters the immune function of astronauts in a manner similar to natural ageing, highlighting the potential impact of spaceflight on immune ageing processes in astronauts.

### Microbial associations with ageing across diverse body sites

Next, we investigated the causal effects between microbiota and ageing using Mendelian randomization. We collected GWAS data from Mibiogen, CNGB, and the IEU‐biobank, covering microbiota data from the gut, oral cavity, and skin [10, 11] (Figure S3A,B).

Using two‐sample Mendelian randomization (TSMR), we identified significant associations between specific microbiota and ageing. In the gut microbiota, one order and three families show correlations with ageing. TSMR analysis also revealed three genera (*Eubacterium rectale, Clostridium innocuum*, and *Enterorhabdus*) that were significantly positively correlated with ageing (Figure 1F), whereas one genus (*Holdemania*) is significantly negatively correlated with ageing (Figure 1F, Table S4). Many of these genera are previously reported to be associated with ageing. In the skin microbiota, specific taxa such as the Gammaproteobacteria, *Micrococcus, Veillonella*, *Cloacibacterium*, *Anaerococcus*, and *Epidermidis* showed significant correlations with ageing (Figure 1F, Table S4). In the oral microbiota, 1 order, 4 families, 3 genera, and 69 species‐level taxa exhibited a strong association with ageing (Figure S3C–E, Table S4).

In summary, TSMR revealed significant associations between microbiota and ageing. However, the GWAS data did not cover all taxonomic levels and species, suggesting the need for further comprehensive studies.

### Microbial shifts in gut, oral, and skin tissues during ageing

We performed a comprehensive metagenomic study to elucidate how age‐related changes in microbial abundance and composition across the gut, oral cavity, and skin might impact the ageing process (Figure 1G,H, Figure S4A,B, Table S5).

In the gut, phylum‐level abundance analysis showed that five phyla‐Pseudomonadota, Bacillota, Euryarchaeota, Actinomycetota, and Thermodesulfobacteriota had higher abundances (Figure S4C). Notably, Pseudomonadota also had the highest abundance in the oral cavity and skin tissues (Figure S5A,B, Table S6). The differential abundance analysis at the phylum level shows that the skin contains the most differential microbiota, and the only phylum with consistent changes across all three tissues is Myxococcota (Figure S4D). Species‐level analysis revealed that in the gut, high‐abundance species were mainly concentrated in Bacillota, with *Faecalibacterium prausnitzii* being the most abundant (Figure S4E). The analysis of alpha diversity (Shannon index) and beta diversity (Bray–Curtis distance) across body sites showed that only skin microbiota diversity was strongly correlated with age (Figure S4F–K).

Differential abundance analysis revealed that in the gut, *Akkermansia muciniphila* was the most significantly downregulated, while *Megamonas funiformis* was the most significantly upregulated in the elderly group (Figure 1I,J, Table S7). The downregulation of *A. muciniphila*, which has been shown to influence gut barrier integrity and modulate immune responses [12], may weaken intestinal mucosal repair and gut metabolic capacity in older adults. Conversely, the upregulation of *M. funiformis*, associated with various diseases, suggests a potential negative impact on health during ageing [13, 14]. In the oral cavity and skin, *Mycobacteriaceae* and *Aeromonas phage CC2* were the most significantly downregulated, while *Ottowia* and *Celeribacter marinus* were the most significantly upregulated (Figure S5C–F). Combined analysis of differential microbiota across the gut, oral cavity, and skin showed consistent upregulation or downregulation of specific species in the elderly group (Figure 1K, Figure S5G, and Table S8). These findings suggest that ageing alters the host microbiota across multiple body sites, leading to changes that may influence the ageing process itself.

### Spaceflight significantly alters microbiota across multiple body sites in astronauts

**Figure 2:**
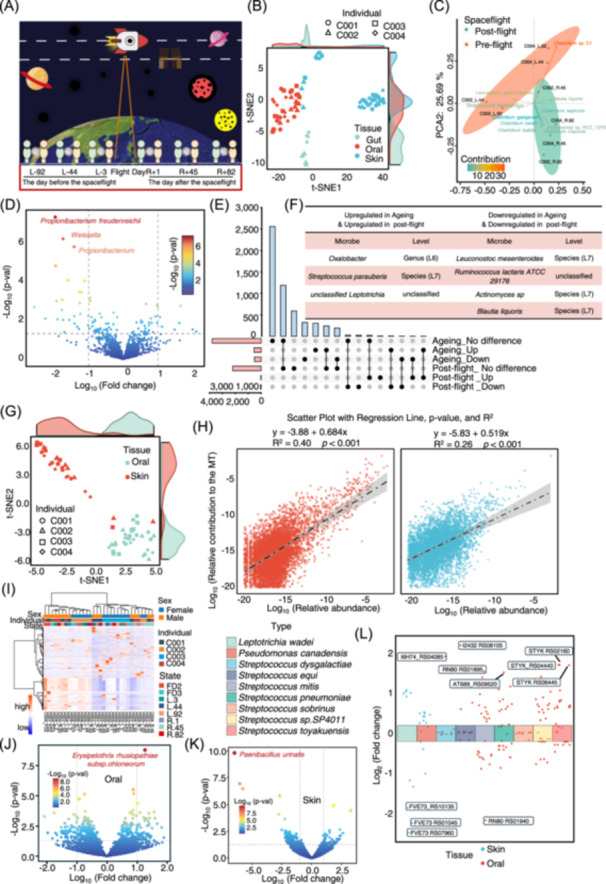
Metagenomic and metatranscriptome data reveal common microbiota changes in the of ageing populations and spaceflight pilots. (A) Schematic overview of data collection and analytical approach. Metagenome‐wide sequencing data collected at six time points surrounding the launch, referred to as L‐92, L‐44, L‐3, R+1, R+45, and R+82, where “L” denotes prelaunch and “R” denotes return (postflight). For example, “L‐92” refers to 92 days before flight. (B) t‐SNE plots illustrating the distribution patterns of samples for each group. (C) Principal component analysis (PCA) illustrating the clustering of gut microbiota in pre‐ and postflight individuals. (D) Volcano plot depicting gut microbial alterations between pre‐ and postflight individuals. The marked microbial communities are the top three significant in the postflight individuals. *Propionibacterium freudenreichii* has been widely reported to regulate the immune system. (E) UpSet plot depicting the distribution of common differential gut microbiota shared between ageing individuals and postflight samples. (F) Table summarizing the common differential gut microbiota observed in both ageing and postflight samples. (G) t‐SNE plots depicting the distribution patterns of samples for each group. (H) Scatter plots illustrating the correlation between oral(left) and skin(right) metatranscriptomes and metagenomics. (I) Heatmap depicting metatranscriptome alignment results to the eggNOG database for oral microbiota. (J) Volcano plot illustrating the relative contribution to metatranscriptome alterations of oral microbiota between pre‐flight and postflight. (K) Volcano plot illustrating the relative contribution to metatranscriptome alterations of skin between pre‐flight and postflight. (L) Scatter plot indicating the species‐level differential analysis results.

To assess microbiota changes across body sites, we collected 72 metagenomic sequencing data from oral, facial skin, and faecal samples of astronauts at different stages of spaceflight [9] (Figure 2A,B, Figure S6A, and Table S9).

Phylum‐level abundance analysis revealed that the five dominant phyla in the gut—Pseudomonadota, Spirochaetota, Bacillota, Planctomycetota, and Campylobacterota—were highly abundant (Figure 2C, Table S10). Among these, Pseudomonadota exhibited the highest abundance in the skin, consistent with findings from the ageing cohort. Species‐level analysis revealed that highly abundant species in the gut were predominantly from Bacillota (Figure S6B), whereas species in the oral cavity and skin were primarily associated with Bacillota and Actinomycetota, respectively (Figure S6C,D). Analysis of alpha and beta diversities revealed no significant correlation between microbiota diversity in the oral cavity, skin, and spaceflight (Figure S6E–H).

The differential abundance analysis revealed that *Propionibacterium freudenreichii*, *Bradyrhizobium*, and *unclassified Bradyrhizobium* were the most significantly downregulated taxa in the gut, oral cavity, and skin, respectively (Figure 2D, Figure S6I). Among them, *Propionibacterium freudenreichii*, a well‐known probiotic, may help regulate inflammatory responses, thus influencing overall health during space missions [15]. In contrast, *Actinomyces massiliensis*, *Mycobacteriales*, and *Weeksella virosa* were the most significantly upregulated in the gut, oral cavity, and skin, respectively (Table S11). Further integrative analysis revealed multiple co‐varying microbial taxa across the three body sites (Figure S7A, Table S11), spanning class, order, family, genus, and species levels (Figure S7B). These findings suggest that spaceflight induces significant alterations in the microbial composition across multiple body sites in astronauts, emphasizing the significant influence of the space environment on the human microbiome.

### Spaceflight modifies age‐related microbiota and offers potential protective strategies

To further investigate the impact of the space environment on astronauts, we performed a combined analysis of the differential microbiota observed after spaceflight and those associated with ageing (Figure 2E). In the gut, we identified that seven potential harmful bacteria significantly increased in the older group; however, these bacteria decreased after spaceflight. In contrast, three potential beneficial bacteria (probiotics) that significantly decreased in the older group increased postflight. Conversely, three potential harmful bacteria that significantly increased in the older group also increased after spaceflight (*Oxalobacter*, *Streptococcus parauberis*, and *unclassified Leptotrichia*). Additionally, four potential beneficial bacteria that significantly decreased in the older group also decreased postflight (*Leuconostoc mesenteroides*, *Ruminococcus lactaris*, *Actinomyces sp*., and *Blautia liquoris*). These findings suggest that targeting the potential harmful bacteria in the former group or supplementing the potential beneficial bacteria in the latter group may provide protective benefits for astronauts (Figure 2F, Figure S7C,D).

To further clarify the relationship between the effects of the space environment and ageing, samples from each body site were divided into three age groups: young, middle‐aged, and older. We applied Weighted Gene Co‐expression Network Analysis (WGCNA) to the microbiota abundance data across these age groups [16], resulting in the identification of multiple independent modules (Figure S8A–P). Modules that showed statistically significant correlations with ageing were designated as ageing‐associated modules (Figure S10A–F, Table S12). To validate the association between these modules and ageing, we employed four machine learning models to predict age groups based exclusively on microbiota abundance from the ageing‐associated modules (Figure S9A). After training, all models demonstrated high accuracy in predicting sample age group using only the abundance data (Figure S9B–J, Figure S10G–J).

We performed an integrated analysis of microbiota strongly associated with ageing and spaceflight, as identified using WGCNA and machine learning (Figure S11A). In the gut, two bacteria negatively correlated with ageing were upregulated after spaceflight, whereas two bacteria positively correlated with ageing were downregulated after spaceflight (Figure S11B). Similarly, ageing‐associated microbiota in the oral cavity and skin exhibited significant changes after spaceflight, and modulating these microorganisms may benefit astronauts during space missions (Figure S11C,D, Tables S13–17).

### Spaceflight alters functional dynamics of microbiota

Metagenomic analyses helped identify microbiota shared between ageing populations and astronauts post‐spaceflight, suggesting that spaceflight affects not only the abundance of these microbes but also their potential function. To elucidate these functional changes, metatranscriptomic analyses were performed (Figure 2G). First, we observed the correlation between the metatranscriptomic and metagenomic datasets from the samples (Figure 2H, Table S18), providing supporting evidence for the consistency of our datasets.

The analysis of the relative contribution to the metatranscriptomic data revealed that *Aeromonas sobria* was the most active species in the oral cavity, whereas *Bartonella grahamii* exhibited the highest transcriptional activity on the skin (Figure S12A, Table S19). To quantify the transcriptional activity of each species, we calculated the relative expression levels by dividing the metatranscriptomic activity by the relative abundance from metagenomic data. We observed that in the oral cavity and skin, Pseudomonadota demonstrated the highest transcriptional activity (Figure S12B).

We performed functional annotation of the metatranscriptomic data to gain insights into the functional status of the oral and skin microbiota (Figure 2I, Figure S12C). Differential pathway analysis showed significant functional alterations post‐spaceflight. In the oral microbiota, disrupted metabolic pathways were enriched in those associated with ageing‐related neurodegenerative diseases. In the skin microbiota, reduced metabolic capabilities in several bacterial taxa were coupled with pathways related to infections and neurodegenerative diseases, whose high‐risk factor is age and which are more prevalent in ageing populations. (Figure S12D,E). These findings suggest that spaceflight may potentially impact human health by altering microbiota function.

Differential expression analyses were performed on the metatranscriptomic data. Co‐altered microbiota identified in the oral cavity and skin during ageing and spaceflight were integrated (Figure 2J–K). This approach helped identify nine species for further investigation (Figure 2L). We mapped the metatranscriptomic data pre‐ and post‐spaceflight to these species to determine their specific gene expression levels. Gene expression analysis revealed significant changes, such as downregulation of *Leptotrichia wadei* genes (FVE73_RS10135, FVE73_RS01045, FVE73_RS07960) in skin microbiota and upregulation of *Streptococcus dysgalactiae* gene I6H74_RS04085 and *Streptococcus equi* gene I2432_RS08105 in oral microbiota (Figure 2L, Table S20). These genes encode ribonuclease P (rnpB) in the respective species. These findings suggest that spaceflight alters microbial activity, potentially affecting astronaut physiology, and underscore the importance of considering both microbial abundance and function in evaluating spaceflight health impacts.

## DISCUSSION

Spaceflight induced significant shifts in microbiota compositions and gene expression, with some changes mirroring natural ageing processes while others diverging. For instance, In the gut microbiota, taxa such as *Oxalobacter, Streptococcus parauberis*, and *unclassified Leptotrichia* exhibited increased abundance in both ageing and post‐spaceflight samples, suggesting their role in ageing‐related processes. In contrast, the skin microbiota showed a distinct pattern, with *Clostridium botulinum, Dermabacter vaginalis*, and *Weeksella virosa* significantly increasing post‐spaceflight, a change not observed in natural ageing. These findings highlight both overlapping and divergent mechanisms between spaceflight‐induced microbiota alterations and natural ageing. The space environment—through factors such as microgravity, radiation, and isolation—induce differential gene expression [5, 17]. Moreover, spaceflight‐related changes in gene expression affected microbial metabolic functions, immune responses, and overall health, particularly influencing T‐cell populations and immune ageing. Collectively, these results provide new insights into how spaceflight influences ageing‐associated microbiota, offering potential targets for intervention to protect astronaut health during extended space missions.

Our study has some limitations. In the single‐cell section, while we observed that spaceflight alters immune function in a manner similar to natural ageing, the specific mechanisms underlying these changes and their direct relationship with microbiota were not explored. In the metagenomic section, the data were sourced from multiple databases rather than from a single individual. Although we controlled for known confounding factors using DESeq.2, we cannot exclude the influence of other potential confounders (e.g., diet, environment, and drug use), which may have impacted the results. In the metatranscriptomic analysis, although we integrated data from the oral and skin microbiota, the absence of gut microbiota data limited our ability to assess functional changes in this critical body site. Additionally, the lack of detailed demographic and metabolic information for the ageing cohort hinders a full assessment of the relationship between ageing, microbiota, and spaceflight effects. Lastly, we did not perform functional validation of the microbiota alterations reported. Future studies with more precise experimental designs, larger cohorts, other diseases (cancer, diabetes, and other chronic diseases), and functional validations are necessary to strengthen our findings. Although we proposed microbiota‐based interventions, their safety, effectiveness, and feasibility should be further assessed through animal models, clinical trials, and in‐flight studies.

Our findings offer preliminary evidence for microbiome‐ and immune‐targeted strategies to protect astronaut health. Further validation is needed. This study advances our understanding of ageing, with implications for healthy ageing both in space and on Earth.

## METHODS

Detailed experimental materials and procedures, including sample collection and processing techniques, and statistical analysis approaches are available in the Supplementary Material.

## AUTHOR CONTRIBUTIONS

**Yuan Sun**: Writing—original draft; validation; investigation; data curation. **Sai Liu**: writing—original draft; visualization; investigation; validation. **Long Chen**: Visualization; formal analysis. **Zheng Zhou**: Investigation; software; validation. **Mengyu Ma**: Visualization. **Jinran Li**: Methodology. **Yi Lu**: Validation. **Yiting Shi**: Methodology. **Tingting Yao**: Validation. **Ruizhi Feng**: Writing—review and editing. **Qiulun Lu**: Writing—review and editing. **Fatimah Qassadi**: Methodology. **Philip M. Williams**: Writing—review and editing. **Tanya M. Monaghan**: Writing—review and editing. **Guangji Wang**: Supervision; resources. **Zheying Zhu**: Project administration; writing—review and editing. **Xinuo Li**: Conceptualization; project administration; supervision; writing—review and editing.

## CONFLICT OF INTEREST STATEMENT

The authors declare no conflict of interest.

## ETHICS STATEMENT

No ethics approval was required for this study because all samples used were publicly available and obtained from open‐access sources.

## Supporting information


**Figure S1:** Immune cell expression patterns associated with ageing and spaceflight.
**Figure S2:** Immune cell expression patterns associated with ageing and spaceflight.
**Figure S3:** Mendelian randomisation analysis of the causal effects of microbiome features on human longevity.
**Figure S4:** Microbial alterations in ageing individuals.
**Figure S5:** Microbial alterations in ageing individuals.
**Figure S6:** Microbial alterations in pre‐ and post‐flight individuals.
**Figure S7:** Microbiota associated with ageing after spaceflight.
**Figure S8:** Key gut microbiota associated with ageing identified using Weighted Gene Co‐expression Network Analysis (WGCNA).
**Figure S9:** Key gut microbiota associated with ageing identified through machine learning.
**Figure S10:** Key oral and skin microbiota associated with ageing identified using WGCNA and machine learning.
**Figure S11:** Microbiota associated with ageing after spaceflight.
**Figure S12:** Metatranscriptomic analyses reveal functional changes of oral and skin microbiota after spaceflight.

**Table S1:** Clinical data of ageing cohort for scRNA analysis.
**Table S2:** Differences analysis results of scRNA in ageing cohort.
**Table S3:** Differences analysis results of scRNA in spaceflight cohort.
**Table S4:** The result of MR.
**Table S5:** Clinical data of ageing cohort for metagenomics analysis
**Table S6:** The OTU of the phyla in ageing cohort.
**Table S7:** Differences analysis results of metagenomics in ageing cohort.
**Table S8:** Microbiota co‐variation across three body sites in ageing individuals.
**Table S9:** Clinical data of spaceflight cohort for metagenomics analysis.
**Table S10:** The OTU of the phyla in spaceflight cohort.
**Table S11:** Differences analysis results of metagenomics in spaceflight cohort.
**Table S12:** The key microbiota of ageing calculated by the WGCNA.
**Table S13:** Common differential oral microbiota between the ageing and post‐flight.
**Table S14:** Common oral microbiota between key microbiota of the ageing and differential microbiota of post‐flight.
**Table S15:** Common differential skin microbiota between the ageing and post‐flight.
**Table S16:** Common skin microbiota between key microbiota of the ageing and differential microbiota of post‐flight.
**Table S17:** Microbial communities with co‐varying trends in the gut, skin, and oral cavity between aging and post‐flight.
**Table S18:** The contribution to the Metatranscriptome at the species level (L7) in two different tissues.
**Table S19:** Differences analysis results of the contribution to the Metatranscriptome in spaceflight cohort.
**Table S20:** The results of DEGs in specific species of two different tissues.

## Data Availability

The data that support the findings of this study are openly available in space at https://github.com/liusai118/space. All original code has been deposited at https://github.com/liusai118/space. Supplementary materials (methods, figures, tables, graphical abstract, slides, videos, Chinese translated version, and update materials) may be found in the online DOI or iMeta Science http://www.imeta.science/.
