# Supplementary material for: Spaceflight redefines ageing‐associated microbiota
Source: Imeta. 2025 Mar 28;4(3):e70023. doi: 10.1002/imt2.70023 (PMC12130553; doi:10.1002/imt2.70023)
Supplement: Supplementary file 1 — Figure S1: Immune cell expression patterns associated with ageing and spaceflight. Figure S2: Immune cell expression patterns associated with ageing and spaceflight. Figure S3: Mendelian randomisation analysis of the causal effects of microbiome features on human longevity. Figure S4: Microbial alterations in ageing individuals. Figure S5: Microbial alterations in ageing individuals. Figure S6: Microbial alterations in pre‐ and post‐flight individuals. Figure S7: Microbiota associated with ageing after spaceflight. Figure S8: Key gut microbiota associated with ageing identified using Weighted Gene Co‐expression Network Analysis (WGCNA). Figure S9: Key gut microbiota associated with ageing identified through machine learning. Figure S10: Key oral and skin microbiota associated with ageing identified using WGCNA and machine learning. Figure S11: Microbiota associated with ageing after spaceflight. Figure S12: Metatranscriptomic analyses reveal functional changes of oral and skin microbiota after spaceflight. [file IMT2-4-e70023-s002.docx]

# Supporting information to Spaceflight Redefines Ageing-Associated Microbiota

Yuan Sun ^1, #^, Sai Liu ^1, #^, Long Chen ^1, #^, Zheng Zhou ^2, #^, Mengyu Ma ^1^, Jinran Li ^1^, Yi Lu ^1^, Yiting Shi ^1^, Tingting Yao ^1^, Ruizhi Feng ^3^, Qiulun Lu ^1^, Fatimah Qassadi ^4^, Philip M. Williams ^4,^ *, Tanya M. Monaghan ^5, 6,^ *, Guangji Wang ^1,^ *, Zheying Zhu ^4,^ *, Xinuo Li ^1,^ *

^1^State Key Laboratory of Natural Medicines, China Pharmaceutical University, Nanjing 211198, China.

^2^Department of Computer Science, RWTH Aachen University, Aachen 52074, Germany.

^3^State Key Laboratory of Reproduction Medicine and Offspring Health, Nanjing Medical University, Nanjing 211166, China.

^4^School of Pharmacy, The University of Nottingham, Nottingham NG7 2RD, UK.

^5^NIHR Nottingham Biomedical Research Centre, University of Nottingham, Nottingham NG7 2RD, UK.

^6^Nottingham Digestive Disease Centre, School of Medicine, University of Nottingham, Nottingham NG7 2RD, UK.

#These authors contributed equally: Yuan Sun, Sai Liu, Long Chen, Zheng Zhou

*Correspondence: [xinuo.li@cpu.edu.cn](mailto:xinuo.li@cpu.edu.cn) (Xinuo Li); [Zheying.Zhu@nottingham.ac.uk](mailto:Zheying.Zhu@nottingham.ac.uk) (Zheying Zhu); [guangjiwang@hotmail.com](mailto:guangjiwang@hotmail.com) (Guangji Wang); [tanya.monaghan@nottingham.ac.uk](mailto:tanya.monaghan@nottingham.ac.uk) (Tanya M. Monaghan); [Pazpmw@exmail.nottingham.ac.uk](mailto:Pazpmw@exmail.nottingham.ac.uk) (Philip M. Williams).

**
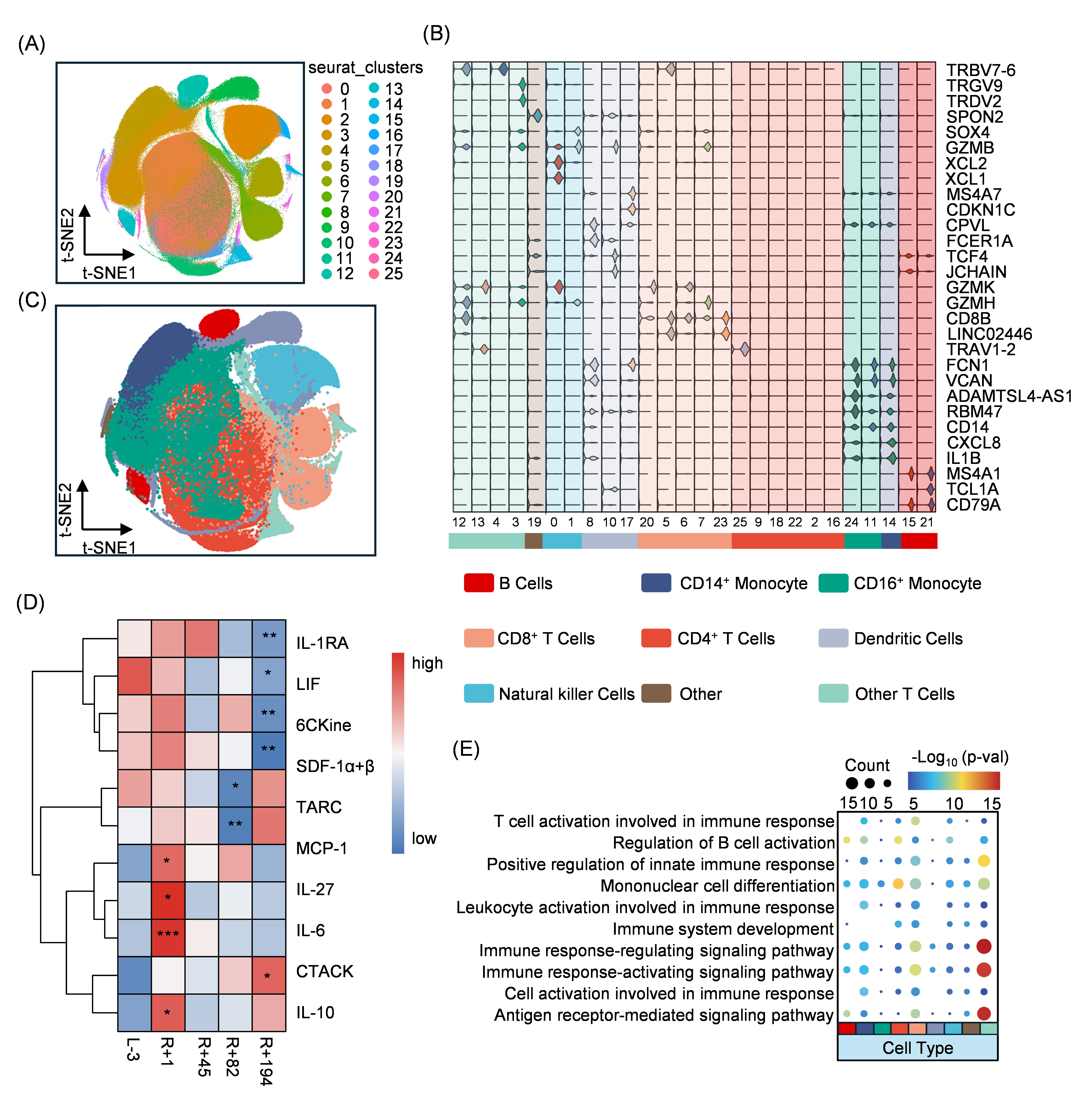
**

**Figure S1. Immune cell expression patterns associated with ageing and spaceflight. (**A). t-SNE plots displaying cell clusters from the ageing cohort detected using 10×Genomics single-cell RNA sequencing (scRNA-seq). (B). Violin plots illustrating the expression of marker genes across 26 clusters. (C). t-SNE plots displaying major immune cell subsets identified in ageing individuals using 10×Genomics scRNA-seq. (D). Heatmap depicting significantly changed biochemicals (cytokines, chemokines, and growth factors) in serum before spaceflight (Pre-flight: mean of L-92, L-44, L-3) and after spaceflight (Immediately Post-flight: R+1, and Long-term Post-flight: R+45, R+82, R+194). (E). Differential Gene Ontology (GO) pathway enrichment analysis of DEGs common to the ageing cohort and post-spaceflight samples.


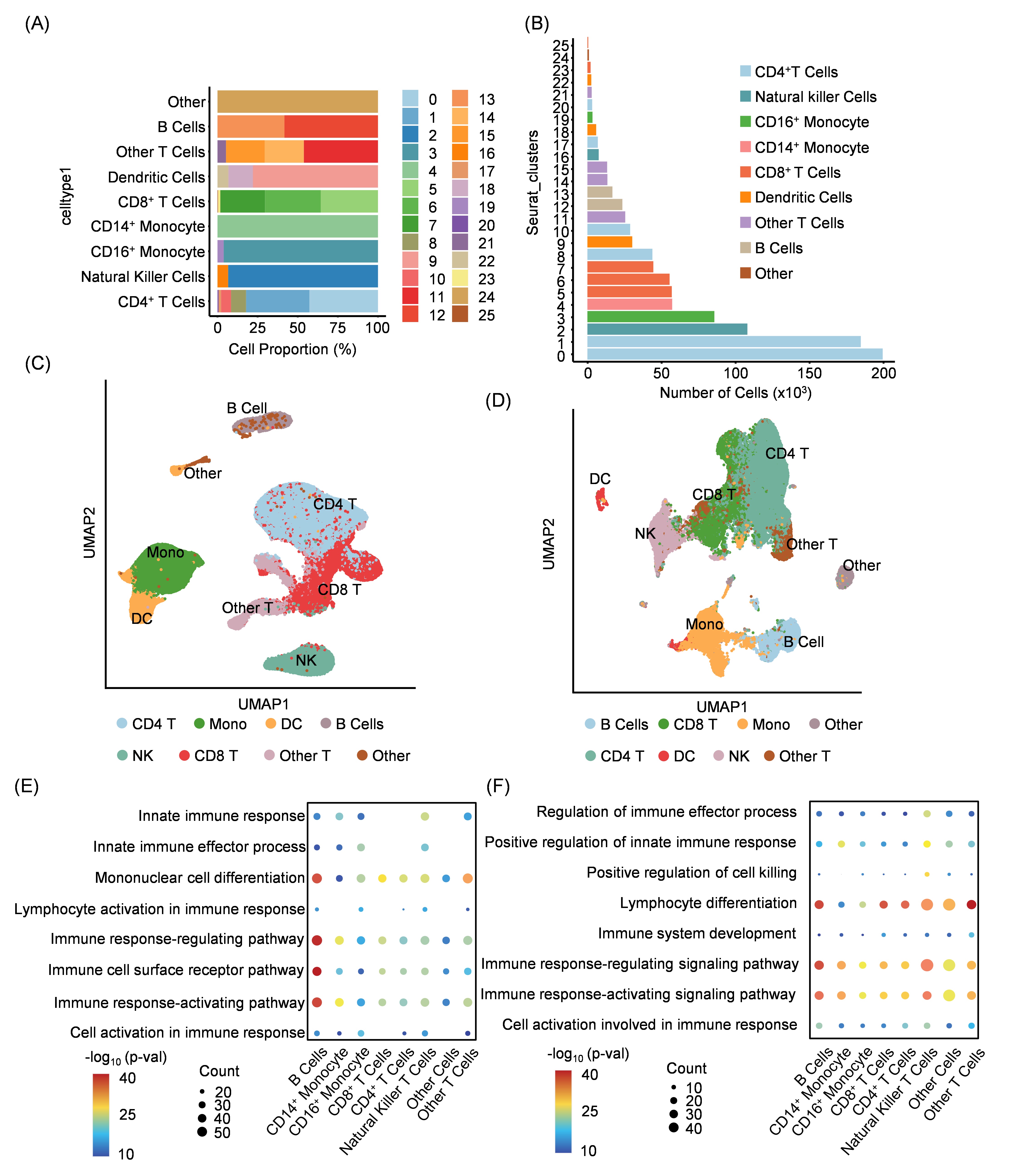


**Figure S2. Immune cell expression patterns associated with ageing and spaceflight. (**A). Bar plots depicting the distribution patterns of diverse immune cells in the ageing cohort. (B). Box plots illustrating the variation in the abundance of diverse immune cells in the ageing cohort. (C). UMAP plots displaying eight immune cell subsets identified using 10×Genomics scRNA-seq in ageing individuals. (D). UMAP plots displaying eight immune cell subsets identified using 10×Genomics scRNA-seq following spaceflight. (E). GO pathway enrichment analysis of DEGs in the ageing cohort. (F). GO pathway enrichment analysis of DEGs following spaceflight.


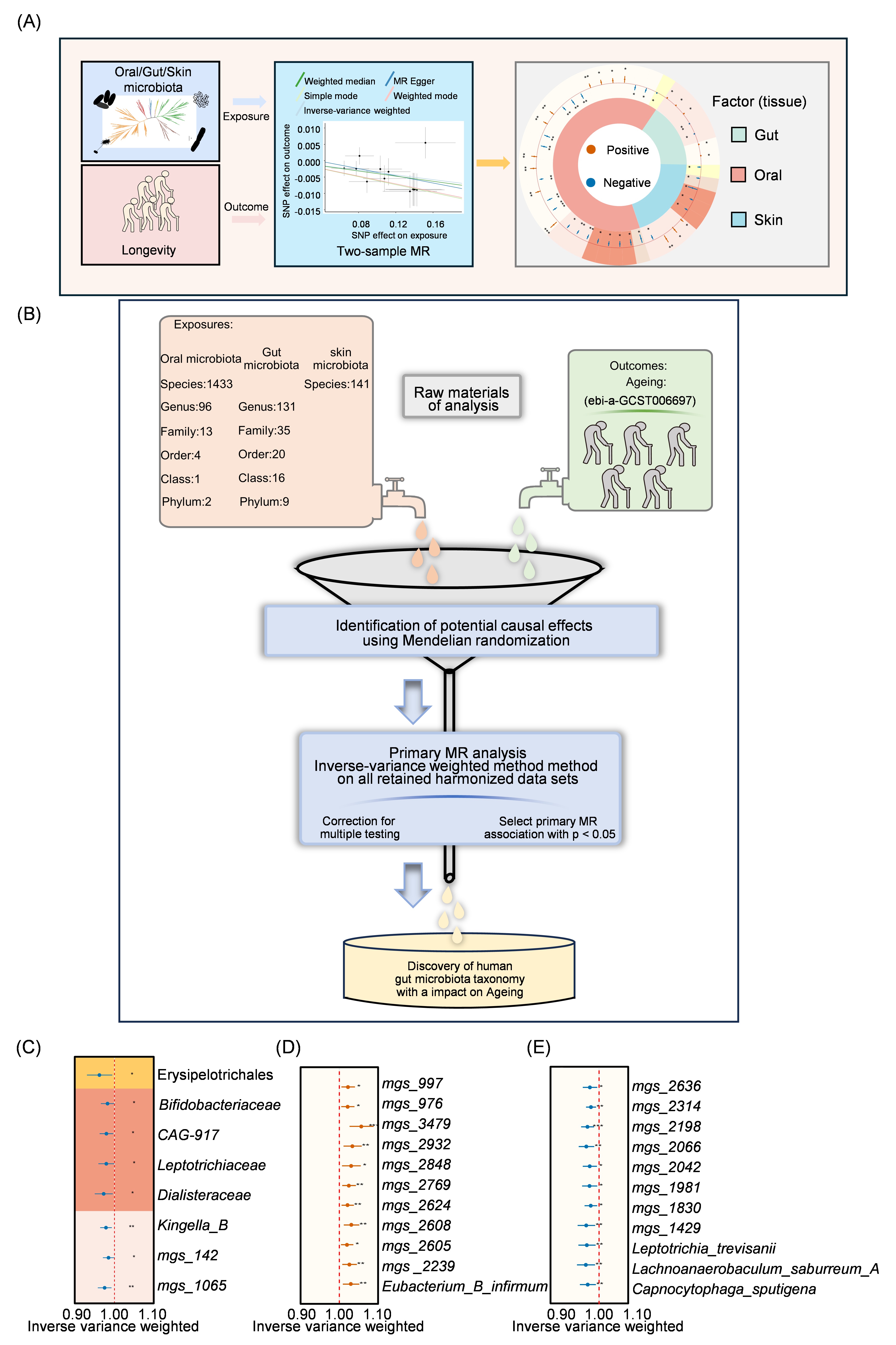


**Figure S3. Mendelian randomisation analysis of the causal effects of microbiome features on human longevity.** (A). Schematic overview of the Mendelian randomisation study design. (B). Workflow pipeline for Mendelian randomization analysis to investigate the causal effects of microbiome features on human longevity. (C). Forest plot showing suggestive causal effects of class-, order-, and family-level oral microbiota on longevity. D-E. Forest plot displaying positive (D) and negative (E) causal effects of species-level oral microbiota on longevity.


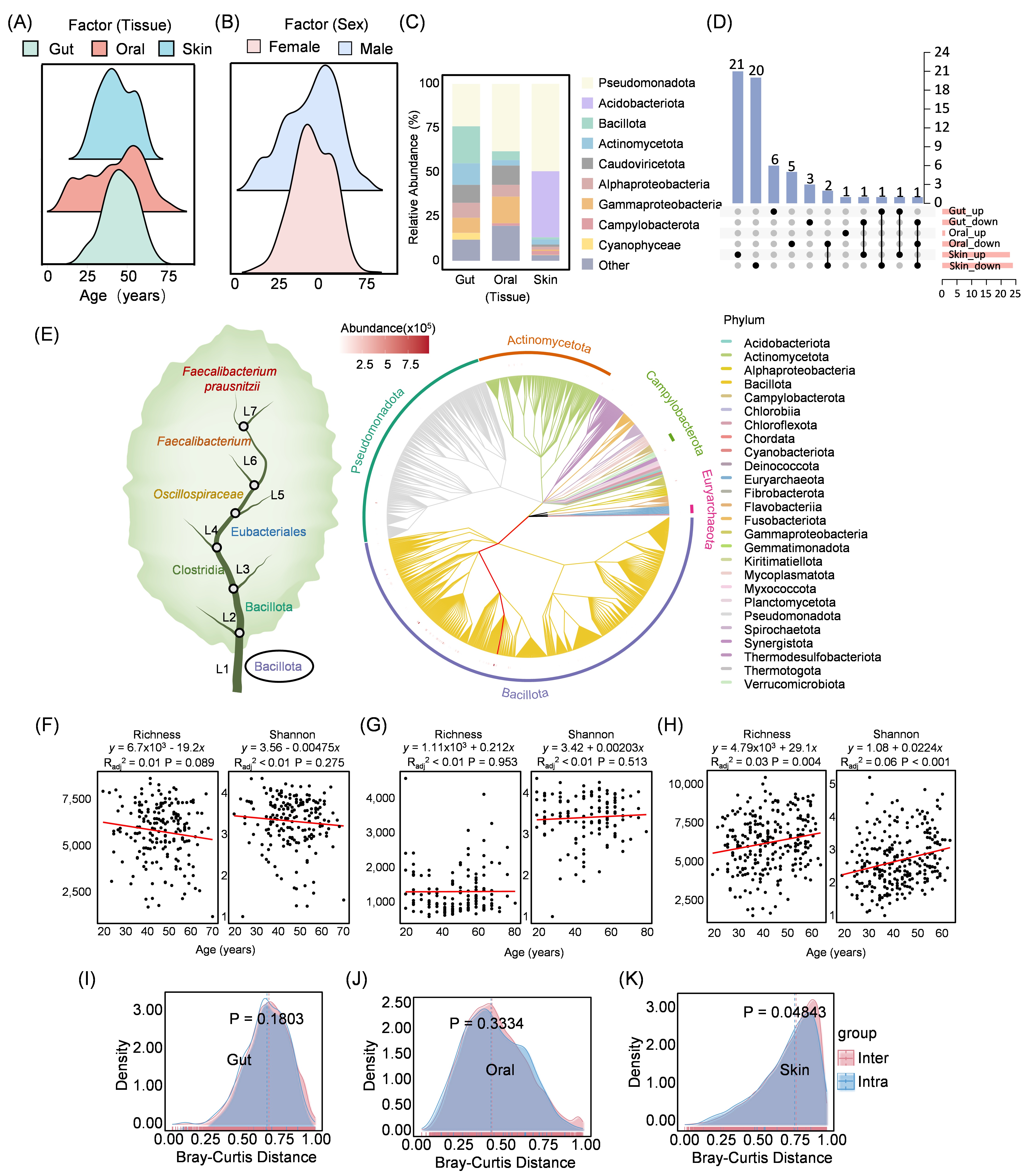


**Figure S4. Microbial alterations in ageing individuals.** (A). Age distribution of Tissue selected for the study. (B). Age distribution of Sex selected for the study. (C). Overview of phylogenetic relative abundances at the phylum level (L2) across three different tissues. (D). UpSet plot depicting the distribution of common differential phyla shared across gut, oral and skin. (E). Phylogenetic tree depicting the 2,000 most abundant gut microbiota. Phylogenetic levels L1–L7 are arranged from the inner to outer layers. (F), (G), (H). Gut (F), oral (G), and skin (H) microbiota based on richness and Shannon diversity indices, grouped by younger and ageing individuals. (I), (J), (K). Beta diversity of gut (I), oral (J), and skin (K) microbiota based on Bray–Curtis dissimilarities, grouped by young and ageing individuals.


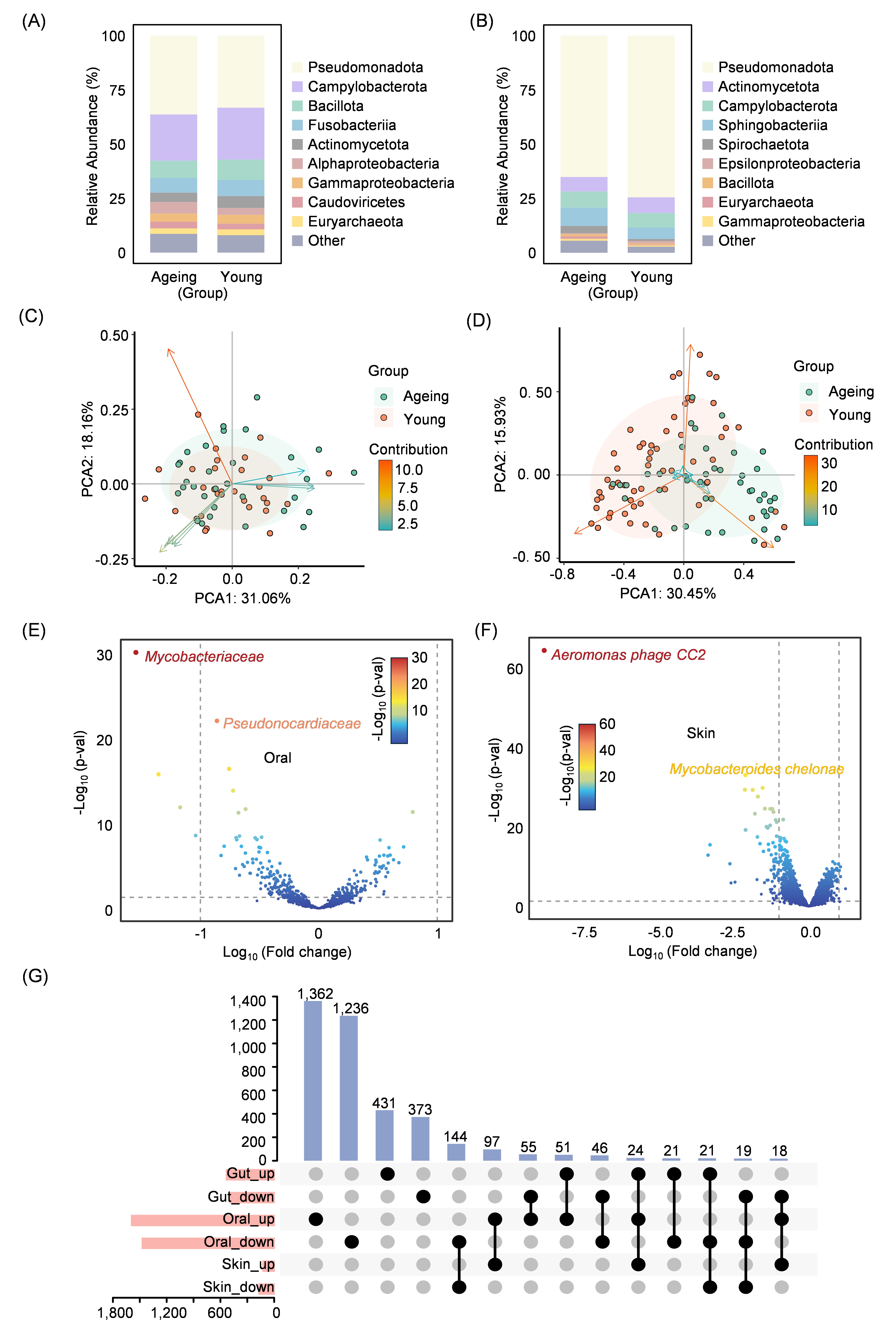


**Figure S5. Microbial alterations in ageing individuals.** Comparison of shotgun sequencing data from samples of young controls and ageing individuals. (A). Overview of phylogenetic relative abundances of oral microbiota at the phylum level (L2) between young and ageing individuals. (B). Overview of phylogenetic relative abundances of skin microbiota at the phylum level (L2) between young and ageing individuals. (C), (D). Principal component analysis (PCA) of oral (C) and skin (D) microbiota in young and ageing individuals. (E), (F). Volcano plot depicting alterations in oral (E) and skin (F) microbiota between young and ageing individuals. (G). UpSet plot illustrating the distribution of differential microbiota across three different tissues between young and ageing individuals.


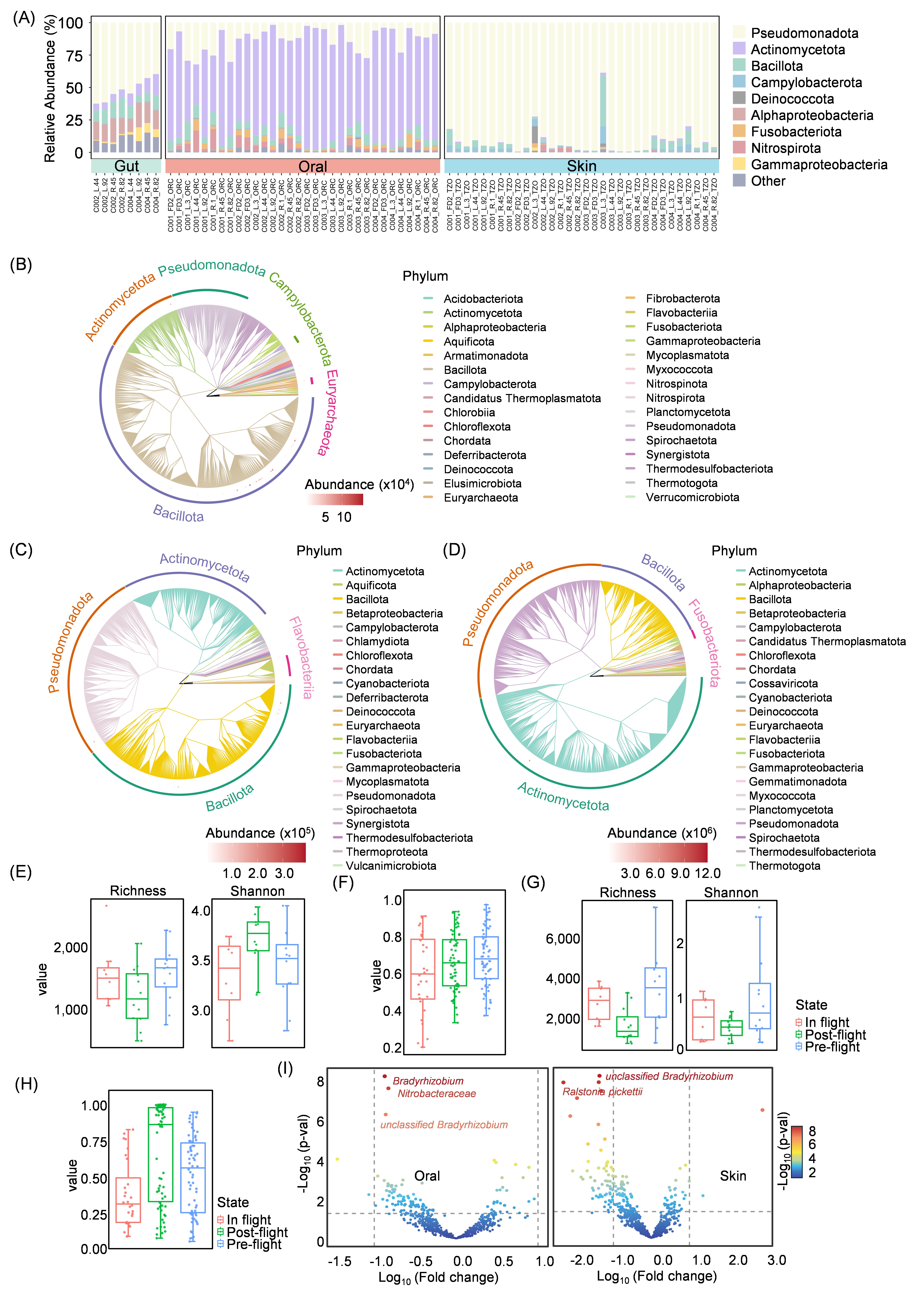


**Figure S6. Microbial alterations in pre- and post-flight individuals.** (A). Schematic overview of data collection and analytical approach. Metagenome-wide sequencing data collected at six time points surrounding the launch, referred to as L-92, L-44, L-3, R+1, R+45, and R+82, where ‘L’ denotes pre-launch and ‘R’ denotes return (post-flight). For example, “L-92” refers to 92 days before flight. (B), (C), (D). Phylogenetic tree illustrating the 1,000 most abundant gut (B) oral (C) and skin (D) microbiota. (E). Alpha diversity of oral microbiome relative abundance data, assessed using Richness and Shannon diversity indices, grouped by pre- and post-flight individuals. (F). Box plots displaying Bray–Curtis distances of oral microbiome samples within each group. (G). Alpha diversity of skin microbiome relative abundance data, assessed using Richness and Shannon diversity indices, grouped by pre- and post-flight individuals. (H). Box plots displaying Bray–Curtis distances of skin microbiome samples within each group. (I). Volcano plots depicting microbial alterations between pre- and post-flight individuals for oral (left) and skin (right) microbiota.


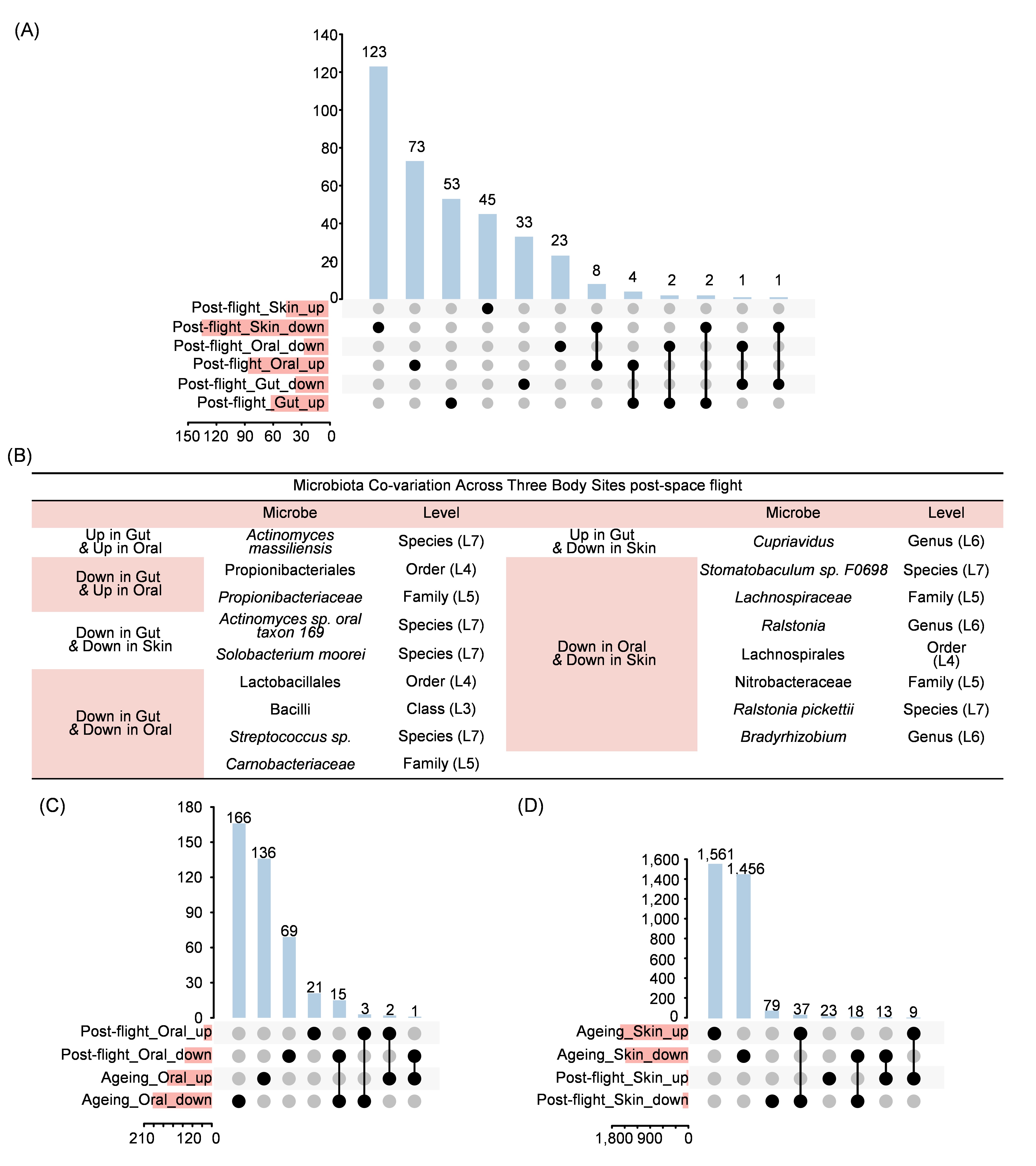


**Figure S7. Microbiota associated with ageing after spaceflight.** (A). UpSet plots illustrating the distribution of differential microbiota across three tissues between pre- and post-flight individuals. (B). Table detailing the common differential microbiota identified between pre- and post-flight individuals. UpSet plot illustrating the distribution of common differential (C). oral and (D). skin microbiota between ageing and post-flight.


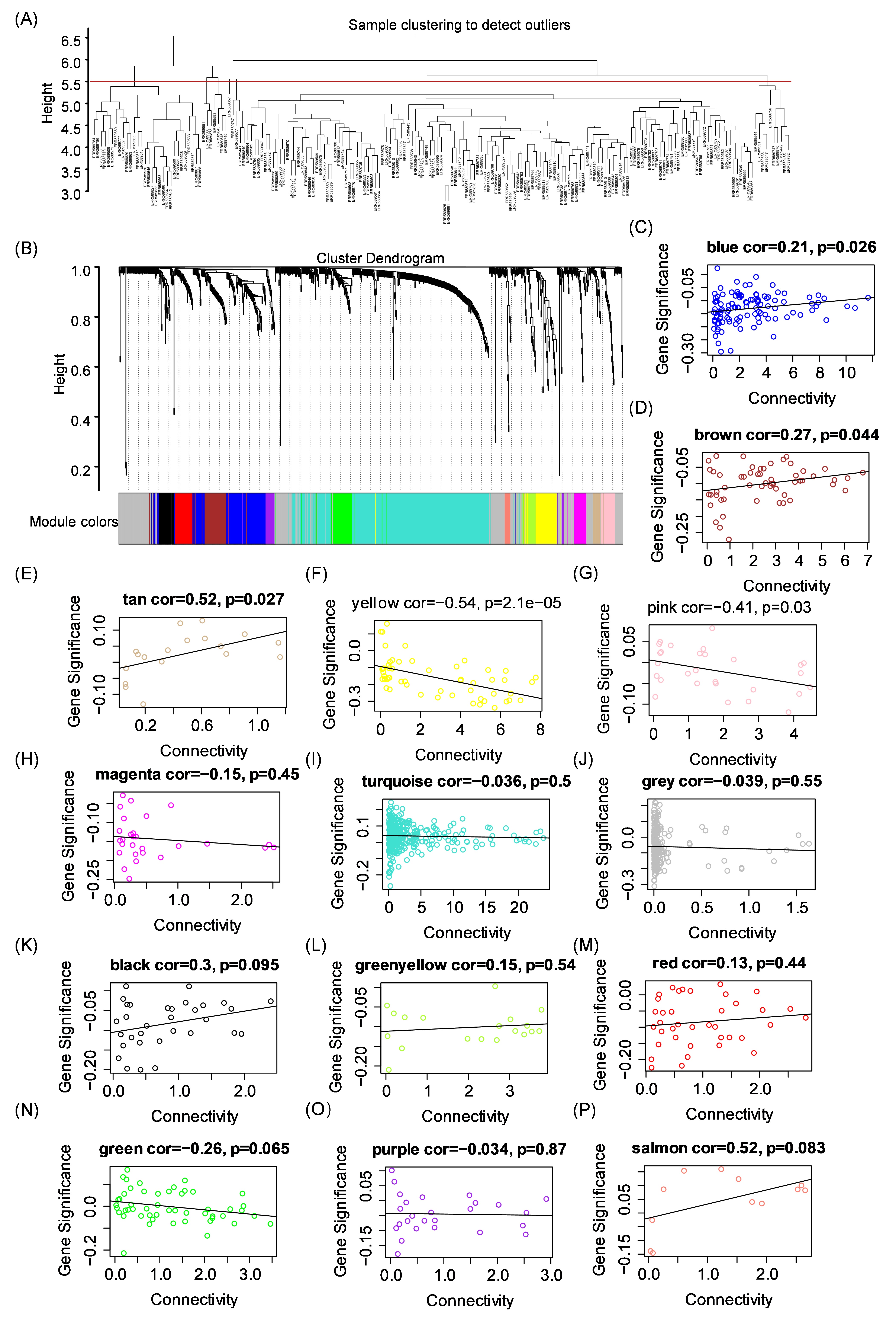


**Figure S8. Key gut microbiota associated with ageing identified using Weighted Gene Co-expression Network Analysis (WGCNA).** (A). Dendrogram illustrating the hierarchical clustering of samples following processing. (B). Composite diagrams displaying the module–trait relationships in WGCNA analysis. (C)–(P). Key WGCNA modules illustrating the correlation between ageing and gut microbial abundance.


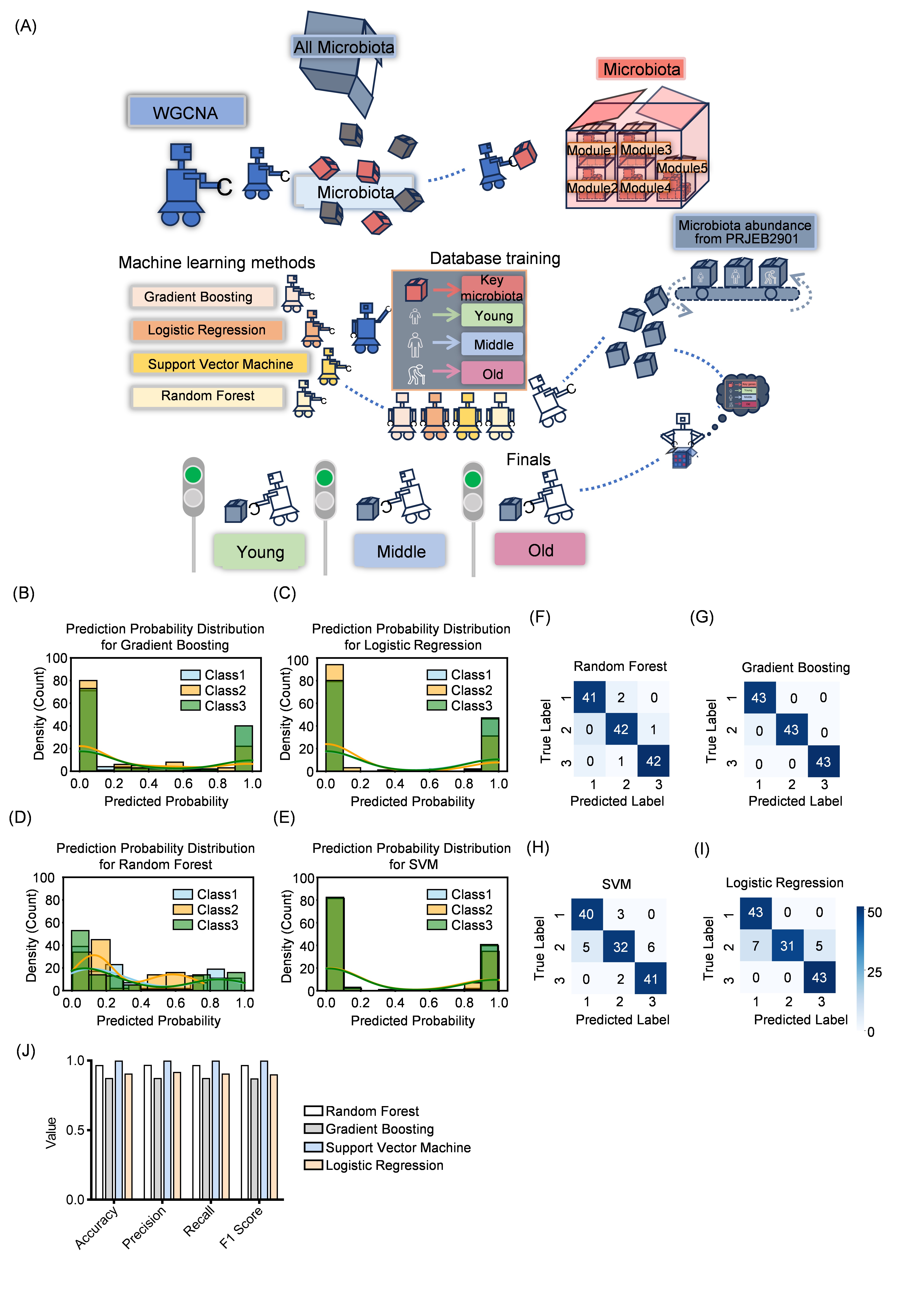


**Figure S9. Key gut microbiota associated with ageing identified through machine learning.** (A). Workflow for machine learning analysis. (B)–(E). Bar plots illustrating the class probability distribution for the hold-out set of the four machine learning models. (F)–(I). Confusion matrices for the hold-out set of the four machine learning models in predicting associations between ageing and gut microbial abundance. (J). Bar chart depicting the comparison of the performance of the four machine learning models using four key metrics: Accuracy, Precision, Recall, and F1 Score.


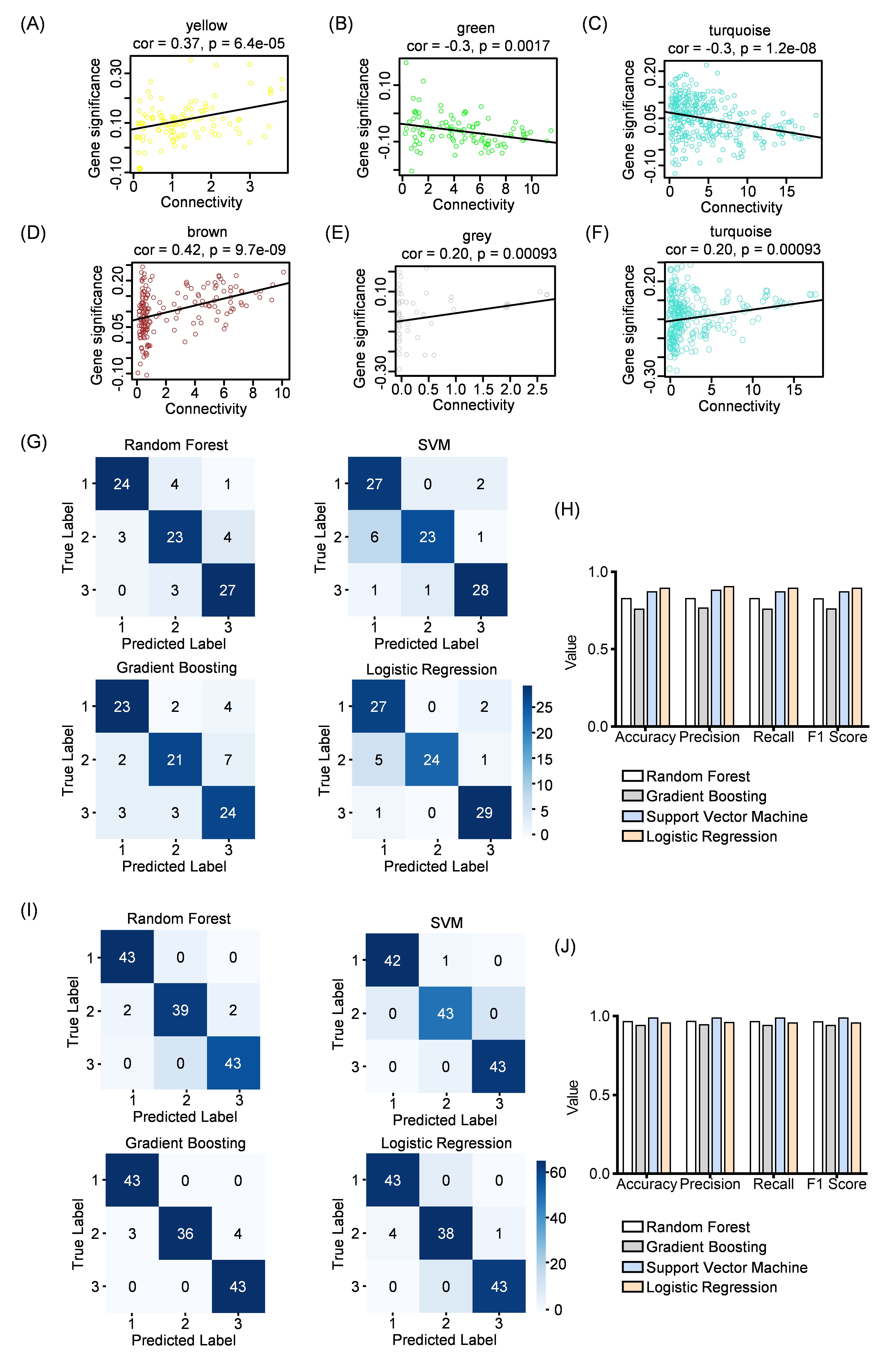


**Figure S10. Key oral and skin microbiota associated with ageing identified using WGCNA and machine learning.** WGCNA modules displaying the correlation between (A)–(D). ageing and skin microbial abundance and (E)–(F). ageing and oral microbial abundance. Performance of four machine learning models in predicting associations between (G)–(H). ageing and skin microbial abundance and (I)–(J). ageing and oral microbial abundance.


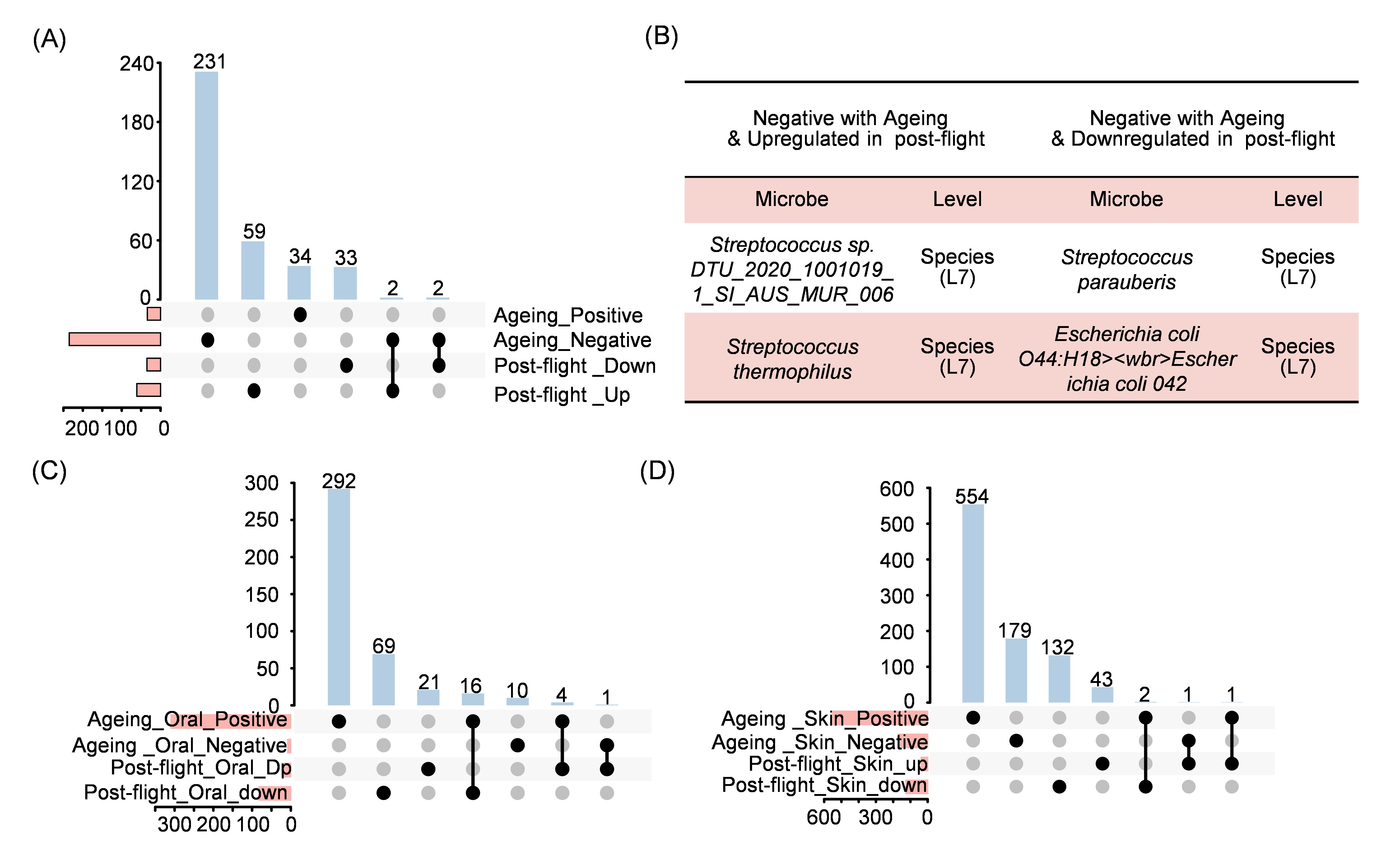


**Figure S11. Microbiota associated with ageing after spaceflight.** (A). UpSet plot illustrating the correlation of gut microbiota between ageing individuals and post-flight samples. (B). Table providing details on the correlational gut microbiota in ageing and post-flight samples. UpSet plot showing the distribution of correlated (C). oral and (D). skin microbiota in ageing and post-flight.

**Figure S12. Metatranscriptomic analyses reveal functional changes of oral and skin microbiota after spaceflight.** (A). Overview of relative contributions to the metatranscriptome at the species level. (L7) in two different tissues. (B). Stacked plot displaying the relative transcriptional activity at the phylum level (L2) in oral(left) and skin(right) microbiota (C). Heatmap depicting metatranscriptome alignment results to the eggNOG database for skin microbiota. (D). Bar plot displaying differential pathways in skin microbiota following spaceflight. (E). Bar plot displaying differential pathways in oral microbiota following spaceflight.

## Methods

### scRNA analysis

The scRNA data of peripheral blood mononuclear cells (PBMC) in ageing cohorts were obtained from Synapse: syn49637038. Seurat (v.4.2.3) was used to conduct the reduction and clustering analysis and harmony (v.1.2.1) was used to eliminate batch effect[1, 2]. Cellmarker2 was used to conduct cell annotation [3]. The DEG was conducted by the Seurat. The GO analysis was conducted by the clusterProfile (v.4.10.1) [4].

### Mendelian Randomization

The GWAS data on gut microbe abundance in five categories were obtained from [*https://mibiogen.gcc.rug.nl/menu/main/home*](https://mibiogen.gcc.rug.nl/menu/main/home). The GWAS data on oral microbe abundance in species were obtained from [*https://db.cngb.org*](https://db.cngb.org) (ID: CNP0001664). The GWAS data on skin microbe abundance in species were obtained from [*https://gwas.mrcieu.ac.uk/*](https://gwas.mrcieu.ac.uk/)(ID: GCST90133164-GCST90133313). Finally, we collected genome-wide association study (GWAS) data comprising 11 phyla, 17 classes, 24 orders, 48 families, 227 genera, and 1,574 species from Mibiogen, CNGB, and the IEU-biobank.

The GWAS data on ageing were collected from [*https://gwas.mrcieu.ac.uk/*](https://gwas.mrcieu.ac.uk/). Initially, we identified all SNPs associated with exposures (p < 5 × 10^-6^). Subsequently, these SNPs underwent clumping using the 1000Genomes Project Phase 3 LD and reference panel to ensure independence of instrumental variables within a 1,000 Kb window and pairwise linkage disequilibrium (LD) r^2^ < 0.001. Instrument strength was quantified using the F-statistic, and the variance explained was measured by r^2^ [5].Variant harmonization was performed by aligning the effect allele betas across different studies using the TwoSampleMR (v.0.4.2.6) package [6]. MRInstruments (v.0.3.2), gwasglue (v.0.0.0.90), Plink (v.1.90-b6.21) and TwoSampleMR were used to conduct local MR analysis [7, 8].

We performed primary Mendelian randomization (MR) analyses for each exposure and outcome association. The inverse-variance weighted method was employed as the principal statistical model to assess potential causal associations between gut microbe abundance and ageing. A total of 1,803 analyses were conducted using the TwoSampleMR and MR-PRESSO packages (v 1.0) in R software (v 4.3.2).

### Preprocess

The metagenomic dataset is composed of biological samples with known ages from PRJEB6997, PRJNA217052, and CNP0000635 and conducting rigorous quality control, we obtained microbiota data from 191 gut samples, 138 oral samples, and 264 skin samples. We obtain the metagenomic data of 72 samples and metatranscriptomics data of 63 samples in Inspiration 4 from the [*https://osdr.nasa.gov/*](https://osdr.nasa.gov/) (OSD-572).

These metagenomic and metatranscriptomics data underwent a standardized quality control pipeline prior to downstream analysis. Unless otherwise specified, the software was run with default settings. Initially, fastp [9](v.0.23.4) was employed for quality control of the raw sequence data to eliminate low-quality sequences. To exclude reads potentially contaminated by human DNA, Bowtie2 (v.2.3.5.1) was utilized with parameters set as very-sensitive-local for aligning the clean data against the human genome (GRCh38.p14) [10].

### Abundance analysis

Taxonomic classification and abundance quantification were performed using Kraken2 (v.2.1.3) and Bracken (v 2 .9), respectively [11]. The default Kraken2 standard reference databases were used, encompassing all NCBI-listed taxa including archaea, bacteria, viral genomes, and plasmids from RefSeq up until May 2024. Abundance analysis based on the results obtained from Kraken2 and bracken was conducted using Pavian (v.1.2.1) [12]. α-diversity estimation of microbiota within each sample was determined by calculating Shannon index values as previously described. β-diversity assessment of inter-sample diversity relied on Bray-Curtis distance calculations. Diversity analyses were performed using pctax R package (v.0.1.1, [*https://github.com/Asa12138/pctax*](https://github.com/Asa12138/pctax)).

### Analysis of metatranscriptomics

The metatranscriptomics data assembly tool is the Megahit (v.1.2.9) and we used Mmseqs2 (v.15.6f452) to reduce sequence redundancy cluster [13, 14]. Prodigal (v.2.6.3) was used to conduct gene predictions[15]. Salmon (v.0.13.1) was used to set the index and quantify the gene expression [16]. After this, we used Diamond (v.2.1.9.163) to align the result to the eggnog (v.6.0) database [17, 18]. The R packages ReporterScore (v.0.1.6) was used to conduct the visualization analysis and differential pathways analysis [19].

### Gene Annotation

We used Hisat2 to construct reference genomes for the following bacterial species (with NCBI accession numbers) [20]:

*Leptotrichia wadei* (GCF_007990545.2_ASM799054v2)

*Pseudomonas canadensis* (GCF_030980095.1_ASM3098009v1)

*Streptococcus dysgalactiae* (GCF_016128095.1_ASM1612809v1)

*Streptococcus equi* (GCF_015689395.1_ASM1568939v1)

*Streptococcus mitis* (GCF_001281025.1_ASM128102v1)

*Streptococcus pneumoniae* (GCF_001457635.1_NCTC7465v1)

*Streptococcus* sp. SP4011 (GCF_037076355.1_ASM3707635v1)

*Streptococcus toyakuensis* (GCF_024346585.1_ASM2434658v1).

We then aligned the processed metatranscriptomic data from each sample to the respective reference genomes using HISAT2 with the parameters --very-sensitive (equivalent to --bowtie2-dp 2 -k 50 --score-min L, 0, -1). Following published study [21], differential gene expression analysis for each bacterial population was performed using DESeq2.

### Differential abundance analysis

DESeq2 (version v.1.42.1), based on the negative binomial distribution, was used to analyze the raw counts to obtain the genes that compare the differences in expression between the groups [22]. In this study, we used the R software package DESeq2 for differential abundance analysis to obtain the differential genes between different comparison groups and control groups.

### Weighted gene co-expression network analysis

Weighted gene co-expression network analysis was performed on the R package WGCNA (v.1.72.5) to discover key microflora related to the ageing [23]. In detail, before the analysis, we use the scale function in R to standardize the abundance data 191 samples. The abundance data of 139 samples were used in the WGCNA after quality control with the cluster dendrogram methods. The soft power threshold was set to 5 to arrive at the network adjacency. The Topology Overlap Matrix (TOM) was created using a cut height of 0.1 and a minimum module size of 25. The analysis identified 14 modules of co-expressed analytes, identified with different colors.

### Machine learning

To validate the reliability of the data from the previous steps, we applied machine learning models to the processed dataset. The original dataset consisted of 139 samples with a significant class imbalance (109 samples for label 2, 18 for label 1, and 12 for label 3). To address this imbalance, we employed the Synthetic Minority Over-sampling Technique (SMOTE), which generated a balanced sample set of 327 samples.

After applying SMOTE, the dataset was split into a training set and a holdout set, with 40% of the data reserved for final model evaluation. This holdout set was crucial for assessing the generalizability of the models and ensuring that the outcomes from the previous data processing steps were reliable.

We selected four machine learning classifiers for evaluation: Random Forest, Support Vector Machine (SVM), Gradient Boosting, and Logistic Regression. The use of multiple models allowed us to mitigate the risk of overfitting that might arise from relying on a single model. By evaluating the performance of different models, we aimed to obtain a more comprehensive and robust assessment, ensuring that the predictions were not overly influenced by the biases or limitations of any one model.

We used Hyperopt to calculate the optimal hyperparameters for each machine learning model and applied these hyperparameters in the respective models [24]. The chosen hyperparameters for each dataset are as follows:

Oral Dataset:

- Random Forest: max_depth = 10, min_samples_split = 2, n_estimators = 250
- SVM: C = 16, probability = True
- Gradient Boosting: learning_rate = 0.0896, max_depth = 3, n_estimators = 60
- Logistic Regression: C = 2.0, max_iter = 600, penalty = l2

Gut Dataset:

- Random Forest: max_depth = 50, min_samples_split = 3, n_estimators = 500
- SVM: C = 2, probability = True
- Gradient Boosting: learning_rate = 0.2952, max_depth = 5, n_estimators = 140
- Logistic Regression: C = 2.0, max_iter = 700, penalty = l1

Skin Dataset:

- Random Forest: max_depth = 40, min_samples_split = 2, n_estimators = 50
- SVM: C = 1, probability = True
- Gradient Boosting: learning_rate = 0.2400, max_depth = 3, n_estimators = 50
- Logistic Regression: C = 1.0, max_iter = 600, penalty = l2

Model performance was evaluated using a 5-fold, 2-repeat stratified cross-validation (Repeated Stratified K-Fold Cross-Validation) approach, ensuring consistent class distribution across all folds. The primary evaluation metrics included accuracy, precision, recall, and F1 score. Additionally, model performance was validated on the holdout set to ensure robustness and confirm the reliability of the previous data processing.

The final assessment on the holdout set demonstrated a strong correlation between model predictions and actual outcomes, further validating the data processing steps undertaken in the previous phases.

### Detailed dataset description

The inclusion and exclusion criteria for the datasets used in the analysis section of this article [25-30], as well as the sample collection methods, are as follows:

A. syn49637038:

inclusion and exclusion criteria:

Participants were given a screening questionnaire to establish their health status. Non-smokers without a history of cancer, chronic inflammatory conditions (arthritis, Crohn’s disease, colitis, dermatitis, fibromyalgia, or lupus), or blood-borne infections (HIV, hepatitis B, and C) were included in the study. Subjects who reported cold or flu symptoms in the prior month were excluded.

sample collection:

Peripheral blood (∼ 100 ml) was collected into sodium-heparin BD vacutainer tubes by venous puncture in the morning (7-10 AM) after an overnight fast. Plasma was isolated from the whole blood by centrifugation at 500 × g for 30 min at room temperature. The top plasma layer was recovered, and aliquots were stored at –80 °C. The remaining whole blood was used for PBMC isolation by density gradient centrifugation using Histopaque-1077 (Sigma, 10771) according to the manufacturer’s instructions. Briefly, the whole blood was diluted in a 1:1 ratio with DPBS (Gipco, 14190136) with 2 mM EDTA (Corning, 46-034-CI). The diluted blood was overlaid on Histopaque-1077 and centrifuged at 500 × g for 30 min at room temperature without breaks. PBMC were isolated from diluted plasma - Histopaque interface and washed twice in DPBS 2 mM EDTA. PBMC were cryopreserved in CryoStor CS10 freezing medium (Biolife Solutions, 210502) and stored at –80 °C.

B. PRJEB29015:

inclusion and exclusion criteria:

Participants were aged 18 to 65 years and had normal values on recent screenings for liver and kidney function, routine blood tests, erythrocyte sedimentation rate, fasting blood glucose, blood lipids, and blood pressure. Subjects were excluded if they had a history of chronic serious infection, any current infection, any type of cancer or autoimmune disease. Pregnant or lactating women were excluded. Subjects who had received antibiotic treatment within 1 month before participating in this study were also excluded.

sample collection:

The patients and healthy controls were asked to provide a frozen faecal sample. Fresh faecal samples were obtained at home, and samples were immediately frozen by storing in a home freezer for less than 1d. Frozen samples were transferred to BGI-Shenzhen, and then stored at –80 °C until analysis. A frozen aliquot (200 mg) of each fecal sample was suspended in 250 µl of guanidine thiocyanate, 0.1 M Tris (pH 7.5) and 40 µl of 10% N-lauroyl sarcosine. DNA was extracted as previously described39. DNA concentration and molecular weight were estimated using a nanodrop instrument (Thermo Scientific) and agarose gel electrophoresis, respectively.

C. PRJEB29015:

inclusion and exclusion criteria:

Participants with no major diseases and no mental disorders, and those under the age of 5, were excluded.

sample collection:

Saliva samples were collected in 20% glycerol and frozen within 30 minutes of collection.

D. CNP0000635:

inclusion and exclusion criteria:

Forty-six male and 248 female healthy volunteers, who were 20 to 65 years old, were recruited from the general population in Shanghai between April and May 2017. Medical and medication history was obtained for each individual by questionnaires. Subjects with any history of skin diseases and intake of systemic or local antibiotics in the past 6 months were excluded. To maximize microbial skin load, each subject was instructed to wash the face only with tap water and to refrain from the application of any skin-care or cosmetic products on the sampling day before sampling.

sample collection:

Three skin sites (forehead, cheek, the back of the nose) were sampled for each subject. Study personnel wore sterile gloves for each sample collection. Samples were collected in a temperature and humidity-controlled room at 20 °C and 50% humidity. To obtain sufficient DNA from the three anatomical skin sites, which were low and variable in microbial biomass, and for the sake of establishing uniform standards between samples, a skin area of 4 cm^2^ was swabbed by sterile polyester fiber-headed swabs moistened with a solution of 0.15 M NaCl and 0.1% Tween 20. The sampling regions were swabbed 40 times each. Then, the swab head was fractured, placed in a sterilized 1.5 mL centrifuge tube, and stored at −80 °C.

E. OSD-572:

Saliva collection:

Saliva was collected at the L-92, L-44, L-3, FD1, FD2, FD3, R + 1, R + 45, and R + 82 timepoints using two methods. First, saliva was collected using the OMNIgene Oral Kit (OME-505), which preserves nucleic acids during the ground timepoints. From these samples, DNA, RNA, and protein were extracted. DNA yield ranged from 28.1 to 3187.8 ng, RNA yield from 396.0 to 3544.2 ng (less the two samples had concentrations too low for measurement), and protein concentration from 92.97 to 93.15 ng.

Skin swabs:

Body swabs were collected at all time points. The samples were collected by swabbing the body region of interest for 30 seconds, then placing the swab in a sterile 2D matrix tube with Zymo DNA/RNA shield preservative. For the first two swab locations, the oral and nasal cavity, the swab was placed directly on the body after removal from its sterile packaging. For the remaining body locations, the swab was briefly dipped in nuclease-free, DNA/RNA-free water before proceeding (wet-swab method).

Stool collection:

Crew members isolated a stool sample using a paper toilet accessory (DNA Genotek, OM-AC1). Stool was transferred into and OMNIgeneGUT tube (DNAgenotek, OMR-200) and an OMNImetGUT tube (DNA Genotek, ME-200). Tubes were placed at -80 °C for long-term storage. For nucleic acid extraction, 200 uL of each tube was allocated for DNA extraction with the QIAGEN PowerFecal Pro kit and 200 uL was allocated to RNA extraction with the QIAGEN PowerViral kit. The remaining sample was split into 500uL aliquots and re-stored at −80 °C.

### Statistical Analysis

For the significance of differentially expressed genes in single-cell analysis, we used the Mann-Whitney *U* test (Wilcoxon rank-sum test) provided by Seurat, with *p* value correction using the Benjamini-Hochberg (BH) method to control the false discovery rate (FDR). This method ensures that the proportion of false positives does not exceed the predefined threshold (significance threshold: 0.01). For the Mendelian randomization analysis, we used the Inverse Variance Weighted (IVW) method (significance threshold: 0.05). This method estimates the overall causal effect by combining the effects of multiple instrumental variables through weighted averaging. For microbial abundance differential analysis, we used the Wald test provided by the DESeq2 package, with a significance threshold of 0.05.

## References

1. Satija, Rahul, Jeffrey A Farrell, David Gennert, Alexander F Schier, Aviv Regev. 2015. “Spatial reconstruction of single-cell gene expression data.” *Nature biotechnology* 33: 495-502. <https://doi.org/10.1038/nbt.3192>

2. Korsunsky, Ilya, Nghia Millard, Jean Fan, Kamil Slowikowski, Fan Zhang, Kevin Wei, Yuriy Baglaenko, Michael Brenner, Po-ru Loh, Soumya Raychaudhuri. 2019. “Fast, sensitive and accurate integration of single-cell data with Harmony.” *Nature methods* 16: 1289-1296. <https://doi.org/10.1038/s41592-019-0619-0>

3. Hu, Congxue, Tengyue Li, Yingqi Xu, Xinxin Zhang, Feng Li, Jing Bai, Jing Chen, Wenqi Jiang, Kaiyue Yang, Qi Ou. 2023. “CellMarker 2.0: an updated database of manually curated cell markers in human/mouse and web tools based on scRNA-seq data.” *Nucleic acids research* 51: D870-D876. <https://doi.org/10.1093/nar/gkac947>

4. Yu, Guangchuang, Li-Gen Wang, Yanyan Han, Qing-Yu He. 2012. “clusterProfiler: an R package for comparing biological themes among gene clusters.” *Omics: a journal of integrative biology* 16: 284-287. <https://doi.org/10.1089/omi.2011.0118>

5. Burgess, Stephen, Simon G Thompson, Crp Chd Genetics Collaboration. 2011. “Avoiding bias from weak instruments in Mendelian randomization studies.” *International journal of epidemiology* 40: 755-764. <https://doi.org/10.1093/ije/dyr036>

6. Hemani, Gibran, Jie Zheng, Benjamin Elsworth, Kaitlin H Wade, Valeriia Haberland, Denis Baird, Charles Laurin, Stephen Burgess, Jack Bowden, Ryan Langdon. 2018. “The MR-Base platform supports systematic causal inference across the human phenome.” *Elife* 7: e34408. <https://doi.org/10.7554/eLife.34408>

7. Purcell, Shaun, Benjamin Neale, Kathe Todd-Brown, Lori Thomas, Manuel AR Ferreira, David Bender, Julian Maller, Pamela Sklar, Paul IW De Bakker, Mark J Daly. 2007. “PLINK: a tool set for whole-genome association and population-based linkage analyses.” *The American journal of human genetics* 81: 559-575. <https://doi.org/10.1086/519795>

8. Minelli, Cosetta, Fabiola Del Greco M, Diana A van der Plaat, Jack Bowden, Nuala A Sheehan, John Thompson. 2021. “The use of two-sample methods for Mendelian randomization analyses on single large datasets.” *International journal of epidemiology* 50: 1651-1659. <https://doi.org/10.1093/ije/dyab084>

9. Chen, Shifu. 2023. “Ultrafast one‐pass FASTQ data preprocessing, quality control, and deduplication using fastp.” *Imeta* 2: e107. <https://doi.org/10.1002/imt2.107>

10. Langmead, Ben, Steven L Salzberg. 2012. “Fast gapped-read alignment with Bowtie 2.” *Nature methods* 9: 357-359. <https://doi.org/10.1038/nmeth.1923>

11. Wood, Derrick E, Jennifer Lu, Ben Langmead. 2019. “Improved metagenomic analysis with Kraken 2.” *Genome biology* 20: 1-13. <https://doi.org/10.1186/s13059-019-1891-0>

12. Breitwieser, Florian P, Steven L Salzberg. 2020. “Pavian: interactive analysis of metagenomics data for microbiome studies and pathogen identification.” *Bioinformatics* 36: 1303-1304. <https://doi.org/10.1093/bioinformatics/btz715>

13. Li, Dinghua, Chi-Man Liu, Ruibang Luo, Kunihiko Sadakane, Tak-Wah Lam. 2015. “MEGAHIT: an ultra-fast single-node solution for large and complex metagenomics assembly via succinct de Bruijn graph.” *Bioinformatics* 31: 1674-1676. <https://doi.org/10.1093/bioinformatics/btv033>

14. Steinegger, Martin, Johannes Söding. 2017. “MMseqs2 enables sensitive protein sequence searching for the analysis of massive data sets.” *Nature biotechnology* 35: 1026-1028. <https://doi.org/10.1038/nbt.3988>

15. Hyatt, Doug, Gwo-Liang Chen, Philip F LoCascio, Miriam L Land, Frank W Larimer, Loren J Hauser. 2010. “Prodigal: prokaryotic gene recognition and translation initiation site identification.” *BMC Bioinformatics* 11: 1-11. <https://doi.org/10.1186/1471-2105-11-119>

16. Patro, Rob, Geet Duggal, Michael I Love, Rafael A Irizarry, Carl Kingsford. 2017. “Salmon provides fast and bias-aware quantification of transcript expression.” *Nature methods* 14: 417-419. <https://doi.org/10.1038/nmeth.4197>

17. Buchfink, Benjamin, Chao Xie, Daniel H Huson. 2015. “Fast and sensitive protein alignment using DIAMOND.” *Nature methods* 12: 59-60. <https://doi.org/10.1038/nmeth.3176>

18. Cantalapiedra, Carlos P, Ana Hernández-Plaza, Ivica Letunic, Peer Bork, Jaime Huerta-Cepas. 2021. “eggNOG-mapper v2: functional annotation, orthology assignments, and domain prediction at the metagenomic scale.” *Molecular biology and evolution* 38: 5825-5829. <https://doi.org/10.1093/molbev/msab293>

19. Peng, Chen, Qiong Chen, Shangjin Tan, Xiaotao Shen, Chao Jiang. 2024. “Generalized reporter score-based enrichment analysis for omics data.” *Briefings in Bioinformatics* 25: bbae116. <https://doi.org/10.1093/bib/bbae116>

20. Kim, Daehwan, Joseph M Paggi, Chanhee Park, Christopher Bennett, Steven L Salzberg. 2019. “Graph-based genome alignment and genotyping with HISAT2 and HISAT-genotype.” *Nature biotechnology* 37: 907-915. <https://doi.org/10.1038/s41587-019-0201-4>

21. Jiang, Yue, Xuejian Xiong, Jayne Danska, John Parkinson. 2016. “Metatranscriptomic analysis of diverse microbial communities reveals core metabolic pathways and microbiome-specific functionality.” *Microbiome* 4: 1-18. <https://doi.org/10.1038/s41587-019-0201-4>

22. Love, Michael I, Wolfgang Huber, Simon Anders. 2014. “Moderated estimation of fold change and dispersion for RNA-seq data with DESeq2.” *Genome biology* 15: 1-21. <https://doi.org/10.1186/s13059-014-0550-8>

23. Langfelder, Peter, Steve Horvath. 2008. “WGCNA: an R package for weighted correlation network analysis.” *BMC Bioinformatics* 9: 1-13. <https://doi.org/10.1186/1471-2105-9-559>

24. Bergstra, James, Daniel Yamins, David Cox.2013. Making a science of model search: Hyperparameter optimization in hundreds of dimensions for vision architectures. *International conference on machine learning*:115-123. [https://doi.org/10.5555/3042817.3042832](https://doi.org/10.1016/j.immuni.2023.10.013)

25. Terekhova, Marina, Amanda Swain, Pavla Bohacova, Ekaterina Aladyeva, Laura Arthur, Anwesha Laha, Denis A Mogilenko, Samantha Burdess, Vladimir Sukhov, Denis Kleverov. 2023. “Single-cell atlas of healthy human blood unveils age-related loss of NKG2C+ GZMB− CD8+ memory T cells and accumulation of type 2 memory T cells.” *Immunity* 56: 2836-2854. e2839. <https://doi.org/10.1016/j.immuni.2023.10.013>

26. Zhang, Xuan, Dongya Zhang, Huijue Jia, Qiang Feng, Donghui Wang, Di Liang, Xiangni Wu, Junhua Li, Longqing Tang, Yin Li. 2015. “The oral and gut microbiomes are perturbed in rheumatoid arthritis and partly normalized after treatment.” *Nature medicine* 21: 895-905. <https://doi.org/10.1038/nm.3914>

27. Brito, IL, S Yilmaz, K Huang, L Xu, SD Jupiter, AP Jenkins, W Naisilisili, M Tamminen, CS Smillie, JR Wortman. 2017. “Correction: Corrigendum: Mobile genes in the human microbiome are structured from global to individual scales.” *Nature* 544: 124-124. <https://doi.org/10.1038/nature20774>

28. Li, Zhiming, Jingjing Xia, Liuyiqi Jiang, Yimei Tan, Yitai An, Xingyu Zhu, Jie Ruan, Zhihua Chen, Hefu Zhen, Yanyun Ma. 2021. “Characterization of the human skin resistome and identification of two microbiota cutotypes.” *Microbiome* 9: 1-18. <https://doi.org/10.1186/s40168-020-00995-7>

29. Tierney, Braden T, JangKeun Kim, Eliah G Overbey, Krista A Ryon, Jonathan Foox, Maria A Sierra, Chandrima Bhattacharya, Namita Damle, Deena Najjar, Jiwoon Park. 2024. “Longitudinal multi-omics analysis of host microbiome architecture and immune responses during short-term spaceflight.” *Nature Microbiology* 9: 1661-1675. <https://doi.org/10.1038/s41564-024-01635-8>

30. Overbey, Eliah G, Krista Ryon, JangKeun Kim, Braden T Tierney, Remi Klotz, Veronica Ortiz, Sean Mullane, Julian C Schmidt, Matthew MacKay, Namita Damle. 2024. “Collection of biospecimens from the inspiration4 mission establishes the standards for the space omics and medical atlas (SOMA).” *Nature Communications* 15: 4964. <https://doi.org/10.1038/s41467-024-48806-z>
